# AIDS advocacy and intellectual property regulation in Brazil: information and influence

**DOI:** 10.7448/IAS.17.1.19396

**Published:** 2014-11-12

**Authors:** Elize Massard da Fonseca, Francisco I Bastos

**Affiliations:** 1 Center for Public Administration and Government Studies, Getulio Vargas Foundation, São Paulo, Brazil; 2 Oswaldo Cruz Foundation, Rio de Janeiro, Brazil

Brazil is well-known for its vigorous AIDS advocacy [1, 2]. During the 1990s, AIDS activists in Brazil centred their agenda on demanding the provision of medicines by pressuring the government through the media and courts to gain legal recognition of their rights to health as guaranteed by the 1988 Constitution [3, 4]. After this period of initial confrontation, AIDS activists became a key partner of the Brazilian Ministry of Health (MoH) in developing prevention initiatives for vulnerable populations [4]. However, in the 1990s, important intellectual property (IP) regulatory decisions were taken without the substantial participation of these groups despite the sweeping effects this would have on their agendas. AIDS activism in Brazil has evolved as activists became aware of the effects of this regulation on access to antiretroviral (ARV) treatment. These new dimensions of the relationship between government and civil society are crucial, as access to ARV treatment is now available to all Brazilians living with HIV irrespective of their clinical and/or immunological parameters, and ARV treatment/prophylaxis has been increasingly viewed as a pivotal intervention in the concerted effort to avert new HIV infections [5, 6].

In recent years, AIDS groups have participated actively in pharmaceutical regulatory debates, closely informing decision makers about the highly technical aspects of IP. The Working Group on IP (GTPI), formed by a group of nongovernmental organizations (NGOs) interested in this issue and coordinated by the Brazilian Interdisciplinary AIDS Association (ABIA), has been the most active group within civil society in articulating the IP debates. A recent and important contribution of AIDS advocates is the regulation of Truvada (a combination of the ARVs tenofovir and emtricitabine) [7], which is the only medicine approved and tested in some at-risk patients for the HIV prevention strategy know as pre-exposure prophylaxis [5]. In December 2006, seven AIDS NGOs filed a request with the Patent Office (INPI) opposing the patent application of tenofovir (tenofovir disoproxil fumarate) (Request No. PI9811045-4). They argued that the only active ingredient that acts on the virus is tenofovir, whereas disoproxil assists in fostering the availability of tenofovir to react with the virus, and fumarate increases the stability of the medicines [8]. The patents for both tenofovir and disoproxil expired 15 and 9 years, respectively, prior to this new grant application. The NGOs also argued that the use of fumarate salt is a chemical practice that has been known since 1963. Therefore, Gilead's application would not fulfil the patentability requirements of the Brazilian legislation (article 8 of the Patent Act). In 2008, the MoH declared tenofovir of public interest, which sped up the application revision by the INPI. Gilead's request was denied in April 2008, and its appeal was denied in July 2010 [9], on grounds similar to those argued by the AIDS activists. Tenofovir is now produced by a public laboratory in Brazil.

Although tenofovir and emtricitabine are not protected by patents in Brazil, Gilead filed a patent request for Truvada in 2004, which was also strongly opposed by the GTPI. Truvada was approved by the Health Surveillance Agency in 2012 for clinical trials, and it is not yet authorized for commercialization in Brazil. These events highlight the capacity of activists to provide information on decisions that are highly technical and to collaborate with the INPI in the process of patent approval, rather than just using media exposure to express their criticism. Their intention was to clarify, based on sound evidence, why the INPI should not grant these patent applications.

Another highly controversial issue has been the “pipeline patents” (i.e. the validation in Brazil of a patent issued abroad, ratifying the examination conducted by a foreign patent office). An economic evaluation conducted by the GTPI compared the purchases of four drugs, which are included in a high-cost medicine programme and protected by the pipeline mechanism, with the lowest international price between 2009 and 2010. They found an excess cost of 55 million dollars for these products in Brazil [10]. In 2007, the GTIP presented to the Brazilian Prosecutor a petition claiming the Action of Unconstitutionality (ADI) of articles in the Patent Act that refer to the pipeline mechanism. Five months later, the Brazilian Prosecutor sent the case to the Federal Supreme Court (STF), questioning the constitutionality of this mechanism by arguing that it does not respect the principle of novelty, as those patents were already in the public domain when granted. In 2008, GTIP's demand received the support of the MoH: “From the point of view of the MoH, the pipeline brings prejudice to the development of the country and has a series of impacts on the Brazilian public health” [11]. However, little has changed since then.

Additionally, since 2007, the representatives of GPTI have been participating in public consultations at the Patent Office (INPI). At first, the INPI resisted this collaboration, arguing that these were technical meetings and thus excluding the participation of civil society on the grounds that it should include only pharmacists. There are two topics on the agenda: polymorphs, an important component of the active pharmaceutical ingredients that may affect the manufacturing process; and the “second medical use,” that is, the innovative therapeutic use of medicines already patented [Arguably, both could be classified as incremental innovations of existing products, thus being subject to patent protection]. AIDS activists support the fact that second medical use and polymorphs should not be granted patent protection. The former is due to its lack of novelty, given that what would be protected by a patent is the medical therapeutic indication, not the product. To them, polymorphism is a natural property (discovered via ordinary experimentation) and, thus, cannot be considered a human invention [12]. The GTPI has also been participating in legislative activities to discuss this issue. Bill Law 2511/2007 proposes to eliminate the patent of second medical use and polymorphs, and the group wrote a complementary legal opinion supporting its approval and quoting other countries that have taken similar decisions (e.g. India). Additionally, the group has educated Congress members on the international guidelines provided by the World Health Organization (WHO), which did not recommend patent protection for second use and polymorphs [12]. This illustrates how these activists have been asserting their rights to inform the government on topics sensitive to IP, rather than just pressuring the government for access to medicines.

Finally, regarding the discussions to end the prior consent mechanism (authority of the National Health Surveillance Agency (ANVISA) to review patent applications together with the INPI), the GTPI has petitioned in favour of this institutional arrangement several times. In 2013, Resolution 21/2013 established that prior consent would remain in operation and clarified under which circumstances ANVISA would be called into the patent application process. The GTPI contributed to this discussion in several ways. In November 2009, the GTPI was invited, together with other representatives of research-based pharmaceutical industries, for a public hearing in Congress to debate Bill Law 3709/2008, which discussed the role of prior consent. The GTPI strongly supported that prior consent should remain unaltered, as this is important to keep patent examination standards high and avoid underserved patents [13]. In 2012, the group submitted its opinions on Resolution 21/2013 through an online open consultation with ANVISA.

In summation, contemporary AIDS activism in Brazil combines traditional strategies to voice the demands of activists for prevention and treatment with informational advocacy on regulatory policy. Most of these issues require a sophisticated understanding of pharmacology and law that is naturally well known to manufacturers of pharmaceutical products [14]. However, AIDS activists have shown remarkable ability and expertise with which to bargain on this topic. How these groups contribute to the policy process is not a result of their size or wealth, but their ability to persuade policy makers with information, as well as the receptiveness of government departments to their demands. We call attention to new models of interaction in the regulatory process in Brazil. These activities have resulted in more balanced and democratic decisions on controversial regulatory decisions.

## References

[1] Galvao J (2000). AIDS no Brasil: a agenda de construção de uma epidemia.

[2] Biehl J (2007). Will to live: AIDS therapies and the politics of survival.

[3] Scheffer M, Salazar A, Grou K (2005). O Remédio via Justiça: Um estudo sobre o acesso a novos medicamentos e exames em HIV/aids no Brasil por meio de ações judiciais.

[4] Nunn A (2008). The politics and history of AIDS treatment in Brazil.

[5] Grant RM, Lama JR, Anderson PL, McMahan V, Liu AY, Vargas L (2010). Preexposure chemoprophylaxis for HIV prevention in men who have sex with men. N Engl J Med.

[6] Ministério da Saúde (2013). Protocolo clínico e diretrizes terapêuticas para manejo da infecção pelo HIV em adultos.

[7] Grupo de Trabalho em Propriedade Intelectual (2012). Registro de medicamento anti-AIDS gera nova demanda por informações Rio de Janeiro.

[8] Abia (2006). Patentes: por que o Brasil paga mais por medicamentos importantes para a saúde pública?.

[9] INPI (2010). Relatorio de Exame Tecnico: PI0406760-6.

[10] Grupo de Trabalho em Propriedade Intelectual (2012). Cálculo estimado do prejuízo monetário causado pela compra de quatro medicamentos selecionados protegidos por patentes pipeline.

[11] Estado de Sao Paulo (2008). Ministerio da Saude quer cancelar artigos da legislacao que criou o pipeline e facilitar a producao de genericos contra o Cancer e AIDS.

[12] Reis R, Vieira M, Chaves C, Reis R, Terto V, Pimenta M (2009). Brazil. Access to medicines and intellectual property in Brazil: a civil society experience. Intellectual property rights and access to ARV medicines: civil society resistance in the global south: Brazil, Colombia, China, India, Thailand.

[13] Chaves C (2009). Audiência Pública – PL 3709/08 – CDEIC – Câmara dos Deputados – Posição do Grupo de Trabalho sobre Propriedade Intelectual da Rede Brasileira pela Integração dos Povos.

[14] Broscheid A, Coen D (2003). Insider and Outsider Lobbying of the European Commission an informational model of forum politics in time: history, institutions and social analysis. Eur Union Polit.

